# Characterization of MK_8_(H_2_) from *Rhodococcus* sp. B7740 and Its Potential Antiglycation Capacity Measurements

**DOI:** 10.3390/md16100391

**Published:** 2018-10-18

**Authors:** Yashu Chen, Qin Mu, Kai Hu, Mo Chen, Jifang Yang, Jigang Chen, Bijun Xie, Zhida Sun

**Affiliations:** 1 Natural Product Laboratory, Department of Food Science and Technology, Huazhong Agricultural University, Wuhan 430070, Hubei, China; yashuchen@sina.com (Y.C.); muqing186@126.com (Q.M.); hzau2015hukai@foxmail.com (K.H.); Bijunxie@sina.com (B.X.); 2 Agricultural Bioinformatics Key Laboratory of Hubei Province, College of Informatics, Huazhong Agricultural University, Wuhan 430070, Hubei, China; m134340@sina.cn; 3 College of Biological and Environmental Science, Zhejiang Wanli University, Ningbo 315100, Zhejiang, China; jfkwlq@163.com (J.Y.); genomic@163.com (J.C.)

**Keywords:** isoprenoid quinone, MK_8_(H_2_), *Rhodococcus* sp. B7740 from Arctic ocean, UPLC-HRMS, NMR, antioxidant and antiglycation

## Abstract

Menaquinone (MK) has an important role in human metabolism as an essential vitamin (VK_2_), which is mainly produced through the fermentation of microorganisms. MK_8_(H_2_) was identified to be the main menaquinone from *Rhodococcus* sp. B7740, a bacterium isolated from the arctic ocean. In this work, MK_8_(H_2_) (purity: 99.75%) was collected through a convenient and economic extraction process followed by high-speed countercurrent chromatography (HSCCC) purification. Additionally, high-resolution mass spectrometry (HRMS) was performed for further identification and the hydrogenation position of MK_8_(H_2_) (terminal unit) was determined using nuclear magnetic resonance (NMR) for the first time. MK_8_(H_2_) showed a superior antioxidant effect and antiglycation capacity compared with ubiquinone Q10 and MK_4_. High-performance liquid chromatography–mass spectrometer (HPLC-MS/MS) and molecular docking showed the fine interaction between MK_8_(H_2_) with methylglyoxal (MGO) and bull serum albumin (BSA), respectively. These properties make MK_8_(H_2_) a promising natural active ingredient with future food and medicine applications.

## 1. Introduction

**Figure 1:**
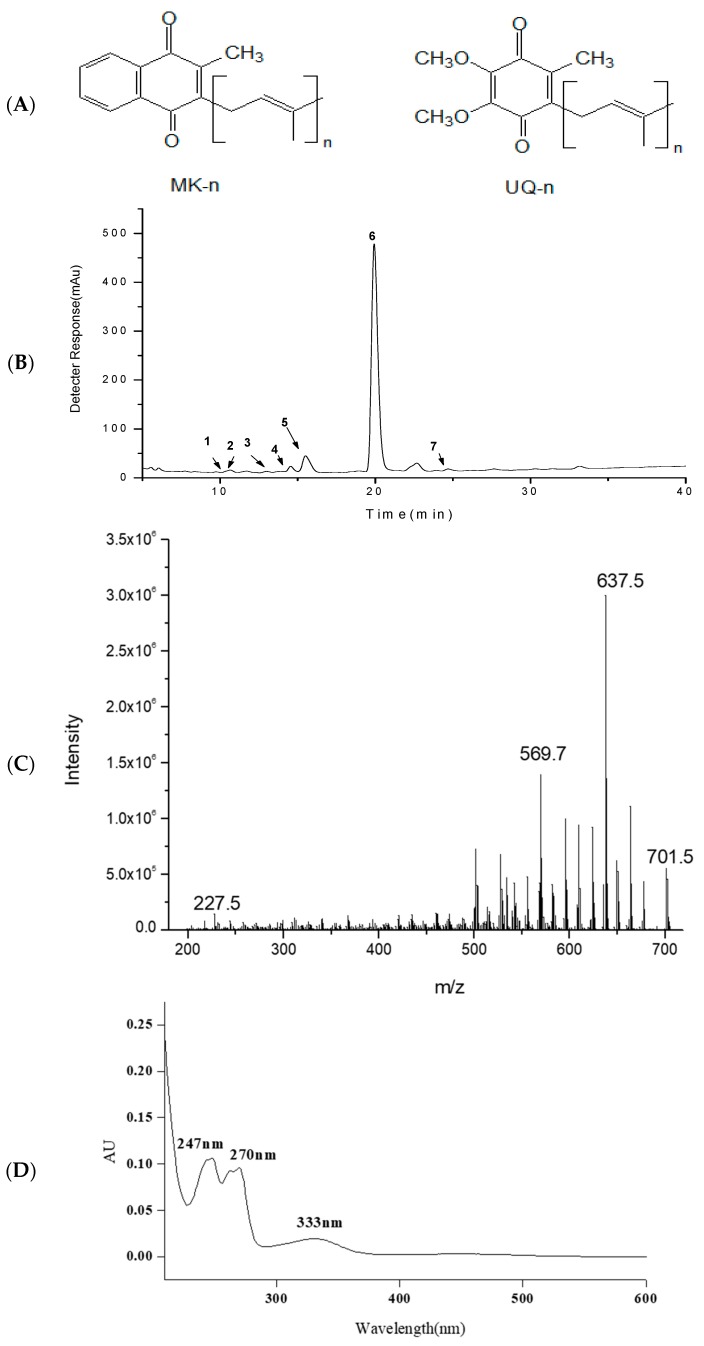
Molecular structure of menaquinone (MK) and ubiquinone (UQ): (**A**) HPLC spectrum of isoprenoid quinones from *Rhodococcus* sp. B7740; (**B**) MS/MS; (**C**) diode array detector (DAD); (**D**) spectrum of MK_8_(H_2_).

Isoprenoid quinone has a characteristic quinone head and an isoprenyl tail which is indispensable in almost all living organisms. Living organisms depend on photosynthetic phosphorylation or oxidative phosphorylation to produce energy, in these processes, isoprenoid quinones play an essential role in transferring protons and electrons between protein complexes. Ubiquinone (UQ) and menaquinone (MK) were identified as two major isopreniod quinone classes (Figure 1) in nature. Generally, natural UQ exist in prokaryotes and eukaryotes, while MK is distributed in archaea and bacteria and serves as an essential vitamin (VK_2_) in the human diet [1].

The structure of isoprenoid quinone gives it its specific functions in organisms. Its lipid-soluble character, given by the isoprenoid side chain, anchors the molecule in membrane lipid bilayers, while the quinone head gives the molecule an electron transfer capacity and defines its redox potential. In mammalian cells, UQ and MK are derived from diet in rather small but crucial amounts. UQ locates itself in the inner mitochondrial membrane and functions in the electron transport chain, and MK functions in blood coagulation, bone metabolism, and cell-cycle regulation etc. [2,3].

Specifically, the increasing demand for skin care products and the public awareness of the health benefits of antioxidants made UQ10 (coenzyme Q10) highly popular as a food supplement [4]. Meanwhile, studies have shown that MK can serve as a treatment for osteoporosis and reducing fractures in the elderly [5,6,7,8]. Empirical researches have shown that MK supplementation might slow atherosclerotic progression and improve cardiovascular function in diseased patients, transplant recipients, and active individuals [9,10,11]. Ongoing researches and hypotheses of the possible functions of MK emerged in large numbers, including treatment for pulmonary elasto-degenerative diseases and mitochondrial pathologies such as Parkinson’s disease and amyotrophic lateral sclerosis [12,13,14]. However, there is still a research gap between the potential applications of MK and its physiological activity, including its molecular mechanism.

As reported, MK_4_, MK_6_, and MK_7_ usually exist in *Flavobacterium*, *Deinococci*, *Elizabethkingia meningoseptica*, and *Bacillus subtilis natto* also contains reasonably high amounts, while MK_8_ was reported to exist in pathogenic bacterium such as *Escherichia coli* [15,16,17,18]. Min at al. used metabolic engineering to increase MK_8_ production, the highest content observed was 55–57 μg MK_8_/g-WCW (wet cell weight) [19]. While in our previous studies, *Rhodococcus* sp. B7740 isolated from 25 m deep seawater at the arctic B77 site was found to produce MK_8_(H_2_) and carotenoids in a rather high amount without gene modification [20]. The structure of MK_8_(H_2_) (especially the hydrogenation position) was not ensured in previous studies. Compared with MK_4_, the longer isoprenoid side chain of MK_8_(H_2_) extends the conjugate structure which may positively affect biological activity [21]. Additionally, hydrogenation could give the molecule a higher stability in the metabolic process. HPLC-APCI-MS/MS (atmospheric pressure chemical ionization source-mass spectrum), high-speed countercurrent chromatography (HSCCC), HRMS, and NMR were conducted to perform the separation, purification, and further identification of MK_8_(H_2_). The hydrogenation position of MK_8_(H_2_) from *Rhodococcus* sp. B7740 was first reported in this work.

Meanwhile, the lower redox mid-potential (E_0_) of MK (−74 mV) compared with UQ (+100 mV) might give MK_8_(H_2_) a better antioxidant activity which has never been studied before [22]. Several studies have reported that the antiglycation (inhibiting advanced glycation end product formation) capability of bioactive compounds might be related to the antioxidant ability and the mechanism of scavenging free radicals. Additionally, the active interaction of MK with proteins related with coagulation function, bone metabolism, and cell-cycle regulation, etc., make us wonder if it can interact with proteins in the formation process of advanced glycation end products (AGEs), which lead us to investigate the potential function of MK in the field of antiglycation. Thus, in this work, the antiglycation ability of MK_8_(H_2_) was first measured using three models and also compared with MK_4_ and Q10. Meanwhile, molecular docking and HPLC-MS/MS were performed to explore the mechanisms of the antiglycation ability of MK_8_(H_2_). According to our study, the hydrogenated MK_8_(H_2_) also possesses high stability (acid and heat resistance). Information from both HRMS and NMR confirmed that the saturated bond was located at the end of the isoprene tail. Antioxidant and antiglycation measurements results showed the superior biological activities of MK_8_(H_2_) and its promising application in the field of medicine.

## 2. Results and Discussion

### 2.1. HPLC-APCI-MS/MS Analysis of MKs

The MKs extracted from *Rhodococcus* sp. B7740 using the acid-hot method were primarily identified based on the combined information obtained from HPLC-DAD-MS/MS and data from published references [23]. The main MKs identified are presented in Table 1. The chromatograms of the MKs monitored at 240 nm are presented in Figure 1B.

According to Table 1, the MKs of *Rhodococcus* sp. B7740 were mainly MK-7, MK-8, and MK-9, which contained 7, 8, and 9 isoprene units, respectively. Among these, Peak 6 is primarily identified as MK_8_(H_2_) with the protonated molecule [M + H]^+^ at *m*/*z* 719, fragment ion at *m*/*z* 701, 637, and 227 (Figure 1C) and a characteristic menaquinone UV spectrum (Figure 1D). As shown in Figure 1B, MK_8_(H_2_) (peak 6, 1025–1075 μg/g-WCW) was the main compound of the extracted MKs. Additionally, the acid-hot extraction method showed that MK8(H_2_) was stable under strong acidic conditions (2 mol/L) at 80 °C after 100 min.

### 2.2. HSCCC Purification and URMS (Ultra-High Mass Spectrum) Analysis of MK_8_(H_2_)

**Figure 2:**
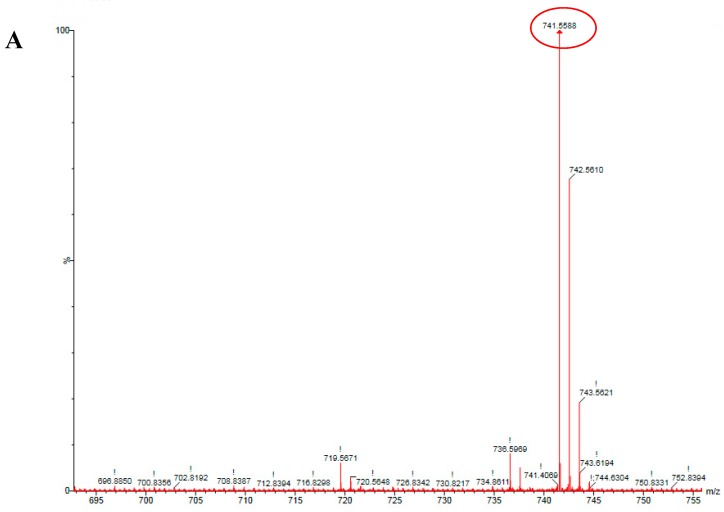

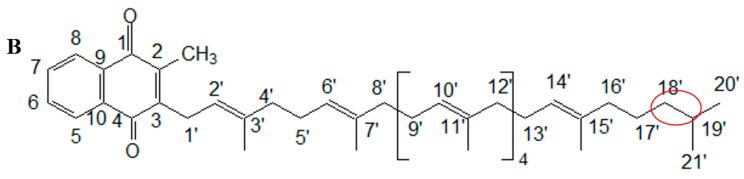
High-resolution mass spectrometry (HRMS) image of: (**A**) MK_8_(H_2_) ((MK_8_(H_2_) + Na^+^) ion); (**B**) molecule structure of MK_8_(H_2_).

Since Peak 6 was the main compound of the MKs from *Rhodococcus* sp. B7740 (Figure 1B), HSCCC was used to purify and collect it. As shown in Figure S1, peak 6 was outflowing and collected during 135–155 min (purity: 99.75%, 807–844 μg/g-WCW). As shown in Figure 2A, the accurate hydrogen molecular ion mass of purified peak 6 was measured by ESI-QTOF-HRMS. Peak 6 with an accurate mass value [M + Na]^+^ at 741.5588 Da (error: 0.13 ppm) was identified as MK_8_(H_2_) (Figure 2B).

### 2.3. NMR Analysis of MK_8_(H_2_)

With the combined information of HPLC-DAD-MS/MS and HRMS, the main compound of MKs from *Rhodococcus* sp. B7740 was identified as MK_8_(H_2_). The MK_8_(H_2_) was speculated to be formed with the addition of two hydrogen from MK_8_. To further analyze the structure of MK_8_(H_2_), especially the position of the two added protons, the ^1^H NMR, ^13^C NMR, HSQC spectra are presented in Figure S1B–D, respectively.

According to Table 2, the chemical shifts at 8.08 ppm, 7.68 ppm, 7.67 ppm, and 8.07 ppm were established as being ArH which were four aromatic protons in the quinoid ring (C-5, C-6, C-7, and C-8, respectively). The δ values of ArH coincide with the typical menaquinone proton NMR values [24]. A chemical shift at 2.19 revealed the presence of Ar–CH_3_ (C-2–CH_3_). A chemical shift at 5.34 revealed the presence of –C=C–(C-2′), which showed a shift toward the low field due to the influence of the aromatic ring. Likewise, a chemical shift at δ 3.38 revealed the protons at C-1′ with the influence of the aromatic ring. Complex absorptions were observed in the δ 3.60–3.75 (protons at C-4′, C-5′, C-8′, (C-9′, C-12′)4, C-13′, C-16′) and δ 5.08–5.12 (protons at C-6′, (C-10′)4) regions due to the presence of six isoprene units. The complex absorptions at δ 0.90–1.48 revealed the six methyl attached to the isoprene units. The chemical shifts at 5.00 ppm were assigned tothe protons at C-14′. The spectra revealed two complex absorptions at δ 0.91–1.20 (C-17′, C-18′) and δ 1.50–2.09 (C-19′). Additionally, a complex absorption at δ 0.82–0.89 revealed the two CH_3_ at the end of the carbon chain, demonstrating the terminal unit was saturated.

Similar analysis was applied to the ^13^C NMR data of MK_8_(H_2_). Since the chemical shifts of MK_8_(H_2_) are listed in Table 2, only some typical ^13^C chemical shifts are discussed here. The chemical shifts at 185.4 ppm, 143.43 ppm, 146.05 ppm, 184.50 ppm, 126.20 ppm, 134.87 ppm, 133.34 ppm, 126.12 ppm, 131.25 ppm, and 132.17 ppm were sighed as ten aromatic carbon atoms in the quinoid ring. These δ values were consistent with the published articles which indicated the existence of the typical menaquinone structure [16]. Chemical shift at 19.60 and 14.20 were assigned as C20′ and C21′, two methyl at the end of the long carbon chain, respectively.

The HSQC spectra (Figure S1C) were presented to further illustrate the position of added protons of MK_8_(H_2_). As shown in Figure S1C, the correlation of δC 19.6 ppm and δC 0.87 ppm and the correlation of δC 14.2 ppm and δC 0.89 ppm revealed the two CH_3_ at the end of the carbon chain. The combined information from^1^H NMR (δ 0.82–0.89), ^13^C NMR (chemical shift at 19.60 and 14.20), and the HSQC spectrum demonstrate that the terminal unit was saturated. The stability of MK_8_(H_2_) is speculated to increase with two more protons at the end of its isoprene tail compared with MK-8.

### 2.4. Antioxidative Effect and Antiglycation Capability

The antioxidant activity of MK_8_(H_2_) was measured and compared with MK_4_ and Q10 using DPPH assay. The scavenging DPPH ability of three quinones increased alongside the increase in concentrations (Figure S2A). The IC_50_ of MK_8_(H_2_) was 2.45 mg/mL (3.4 μmol/L), which was larger than that of MK_4_ (0.85 mg/mL, 1.9 μmol/L), indicating a lower inhibiting DPPH effect. The inhibition rate of Q10 at the max detected concentration (7 mg/mL, 8.1 μmol/L) was still under 50%, which might indicate a lower antioxidant ability than MK_8_(H_2_) and MK_4_.

In the BSA-fructose model (Figure S2B), MK_8_(H_2_), MK_4_, and Q10 all presented an inhibitory effect against the formation of AGEs in a dose-dependent mode. The IC_50_ of MK_8_(H_2_) was 5.26 mg/mL (7.3 μmol/L), which was smaller than that of Q10 (20.22 mg/mL, 23.4 μmol/L). The inhibition rate of MK_4_ at the maximum detected concentration was still under 50%. These results exhibited a different trend with the DPPH inhibiting effect measurement, which may indicate that menaquinone and ubiquinone are capable of inhibiting AGEs formation in the BSA-fructose model, which was not related with the antioxidant ability.

Similarly, in the BSA-MGO (methylglyoxal) model (Figure S2C), MK_8_(H_2_) also showed a significant effect in AGEs inhibition, compared with MK_4_ and Q10. The IC_50_ of MK_8_(H_2_) was 9.02 mg/mL (12.6 μmol/L), while the inhibition percentage of MK_4_ and Q10 were both under 50% at the maximum detected concentration.

One of the irreversible glycation processes of the protein was the binding of arginine with MGO [25]. In the arginine-MGO model (Figure S2D), MK_8_(H_2_) also showed a most significant effect on AGEs inhibition, compared with MK_4_ and Q10. The IC_50_ of MK_8_(H_2_) was 8.69 mg/mL (12.1 μmol/L), while the inhibition percentage of MK_4_ and Q10 were both under 50% at the maximum detected concentration.

### 2.5. Analysis of MK_8_(H_2_)-Carbonyl Adducts by LC-MS/MS

Several studies have reported that the antiglycation capability of bioactive compounds might be based on the mechanism of scavenging free radicals and MGO, attenuating its attack to amino acids [26,27,28,29]. In this study, the MK_8_(H_2_)–MGO adduct was investigated by LC-MS analysis.

**Figure 3:**
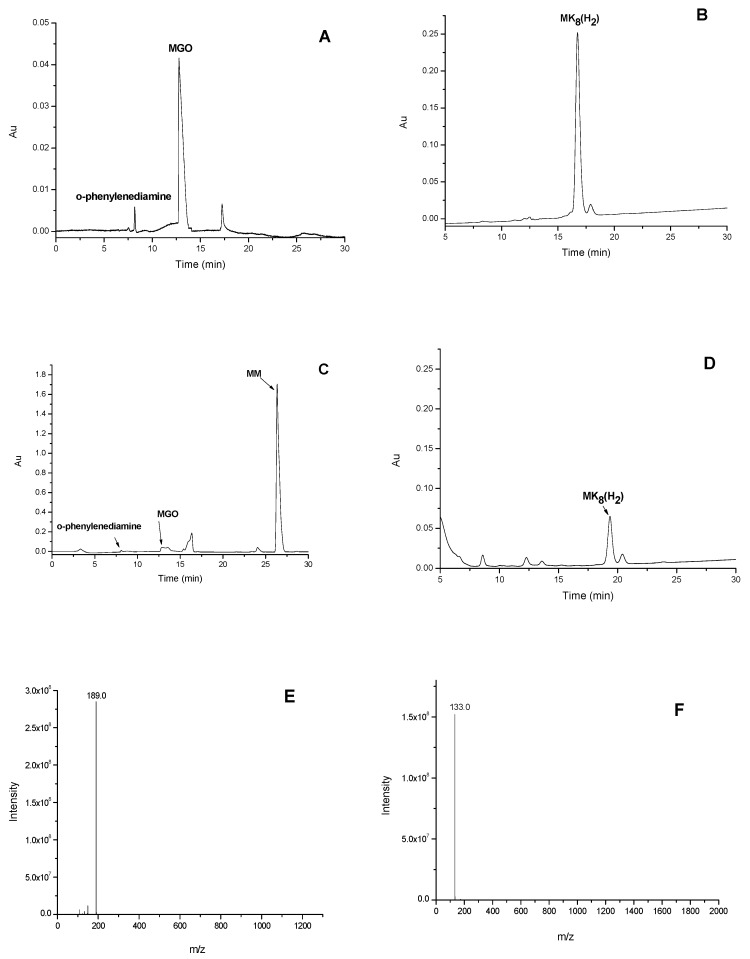
The HPLC spectrum of MGO (**A**) before and (**C**) after incubation. The HPLC spectrum of MK_8_(H_2_) (**B**) before and (**D**) after incubation. (**E**) MS and (**F**) MS/MS spectrum of MK_8_(H_2_)–MGO adduct.

The formation process of MK_8_(H_2_)–MGO conjugate was investigated by HPLC and HPLC-MS/MS analysis. The HPLC spectrums of MK_8_(H_2_), MGO and mass spectra of their conjugates were presented in Figure 3. The MGO molecule (Figure 3A,C) and MK_8_(H_2_)molecule (Figure 3B,D) were both analyzed using HPLC, before and after incubation. After the MK_8_(H_2_) and MGO solutions had reacted, these two molecules were significantly reduced. Additionally, the HPLC-MS/MS were used to detect the MK_8_(H_2_)–MGO conjugate. As shown in Figure 3C, the peak at 26.3 min was observed with the MK_8_(H_2_)–MGO adduct (MM). As shown in Figure 3E,F, MM possessed a protonated molecule [M + H]^+^ at *m*/*z* 189, fragment ion at *m*/*z* 133, which indicated a loss of a fragment ion of MGO (C_3_H_4_O^+^, *m*/*z* = 56). Thus, MM (C_11_H_24_O_2_) was speculated as being a dimer of C_4_H_10_ (one hydrogenated isoprene unit) added with one molecule of MGO.

### 2.6. Docking Studies

**Figure 4:**
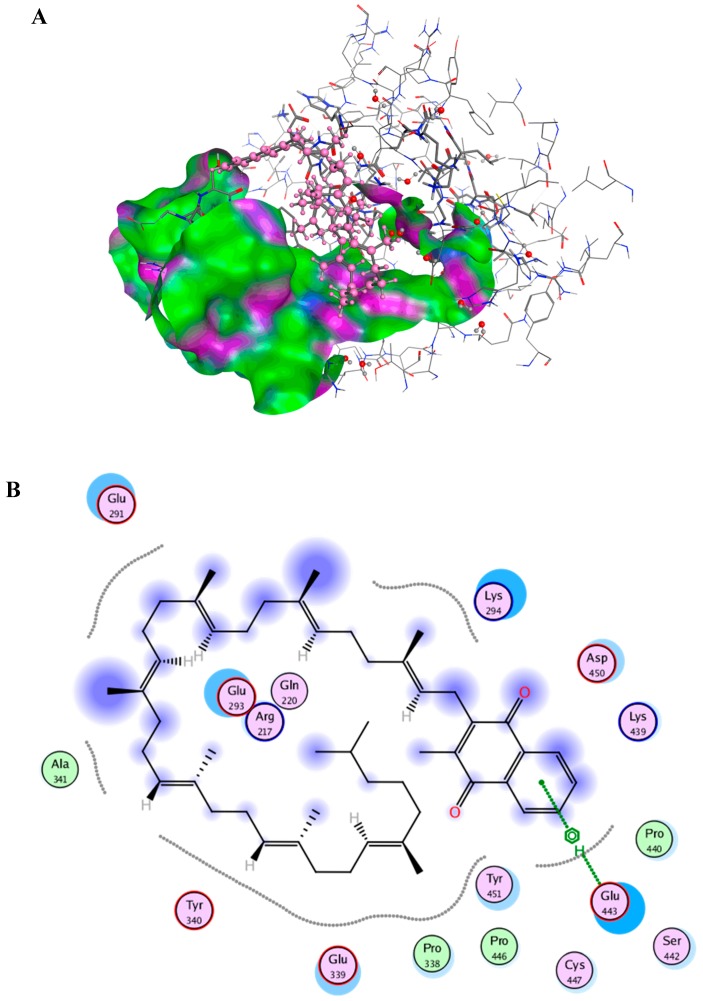

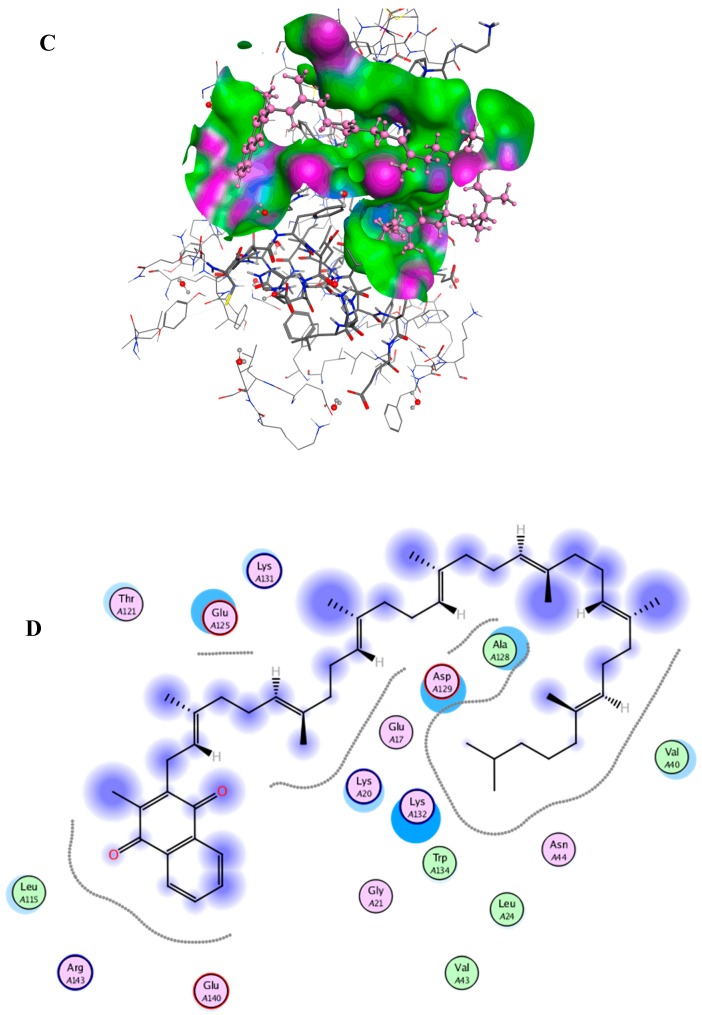
(**A**)Main interaction of MK_8_(H_2_) binding to the BSA at site I; (**B**) Molecular contacts between MK_8_(H_2_) and amino acids of BSA at site I; (**C**) Main interaction of MK_8_(H_2_) binding to the BSA at site II; (**D**) Molecular contacts between MK_8_(H_2_) and amino acids of BSA at site II.

After the docking simulation, the structure of the most likely binding conformation and the 2D interaction diagram of BSA with MK_8_(H_2_) in both site I and site II were captured. The interaction of MK_8_(H_2_) with BSA at site I are presented in Figure 4A,B, which shows that the MK_8_(H_2_) can perfectly fit into site I. The hydrophobic interaction is shown as the most significant interaction pattern in this system. At the same time, the residue Glu443 of BSA is intended to generate a arene-H interaction with MK_8_(H_2_). Figure 4C,D shows the interaction of MK_8_(H_2_) with BSA at site II. As shown in Figure 4C,D, MK_8_(H_2_) also fits well into site II, and the hydrophobic interaction is again the main interaction pattern. The docking studies demonstrated the fine binding between MK_8_(H_2_) and BSA, which may explain the internal mechanism of the antiglycation effect of MK_8_(H_2_) in the BSA-fructose and BSA-MGO model.

Studies focused on menaquinones, especially hydrogenated MK_8_(H_2_), are rather rare. Hence, there is a research niche which is generating great interest. According to our studies, we believe the hydrogenated position (end of the eight isoprene units) and its typical menaquinone head give MK_8_(H_2_) a unique activity including higher stability (acid and heat resistance) and a stable antiglycation ability.

## 3. Materials and Methods

### 3.1. Extraction of MKs

*Rhodococcus* sp. B7740 lyophilized powders (0.1 g) were crushed with 4 ml hydrochloric acid (2 mol L^−1^) and incubated at 80 °C for 100 min after a 15 s vortex. The mixture was centrifuged for 10 min at the speed of 7000× *g* at 4 °C. The residues were retained and resolved in 6 mL of acetone. After a 15 s vortex and a 10 min centrifugation (7000× *g*, 4°C), the MKs were adequately extracted in the supernatant.

### 3.2. LC-MS/MS Analyses of MKs

MKs in acetone were concentrated by rotary evaporation and then resolved by methanol and MTBE (methyl tert-butyl ether) (1:1, *v*/*v*) and filtered using 0.22 μm millipore filter (nylon 66) for further analysis. The LC-APCI-MS/MS analysis was performed according to the previously published article [20].

### 3.3. HSCCC for Purification of MKs

The separation procedure was performed according to the afore mentioned article with a few changes [20]. Briefly, MKs in acetone were concentrated by rotary evaporation and then resolved by the lower phase and then injected into the TBE-300C semi-preparative HSCCC equipment after hydrodynamic equilibrium. The effluent was monitored by a UV-Visible detector at 242 nm.

### 3.4. UPLC-ESI-QTOF-HRMS Analysis of MKs

The purified MK compound was collected with the separation procedure described previously. The UPLC-ESI-QTOF-HRMS analysis was conducted on an Acquity UPLC system (Waters, Milford, MA, USA), equipped with a BEH (bridge hybrid ethyl particle) Shield RP18 column (100 mm × 2.1 mm, 1.7 μm). The mobile phases of the A and B pump were both methanol and the flow rate of the isocratic elution was 0.3 mL/min. The injection volume was 2 µL, and the column temperature was set at 25 °C. Full MS scans were acquired in positive ion mode with a mass range of *m*/*z* 400–1000 at a resolution of 60,000. The other applied HRMS parameters were set as in the published article [20].

### 3.5. NMR Analysis of MKs

^1^H NMR, ^13^C NMR, and HSQC spectra were recorded at 600 MHz on AVANCE III 600 (Bruker, Billerica, MA, USA). Tetramethylsilane (TMS) was used as internal standard. The purified MK was dissolved in 500 μL of CDCl_3_ in a NMR tube (5 mm) for NMR spectrometry [30].

### 3.6. Determination of Antioxidative Effect and Antiglycation Capability

The antioxidant activity of MKs and Q10 were determined by 2,2-diphenyl-1-picrylhydrazyl (DPPH) scavenging activity, which was measured based on the published article [31].

Three models, including the BSA-fructose model, BSA-MGO model, and arginine-MGO model, were used to simulate the process of protein glycation and AGEs production based on the former studies with minor modifications [32]. Onemillilitreof BSA (60 mg/mL in 0.01 mol/L PBS (phosphate buffer saline) pH 7.4) and 1 mL of fructose (1.5 mol/L in 0.01 mol/L PBS, pH 7.4) solution were mixed and incubated with 100 μL of MK_8_(H_2_), MK_4_ or, Q10 solution (2.5, 5, 10, 15, or 20 mg/mL) at 50 °C for 24 h, respectively. Acetone (100 μL) was used as a blank control. To calculate the formation of AGEs, the fluorescence intensities were measured by fluorescence spectrophotometer (F-4600, HIT) equipped with a 1.0 cm quartz cell at an excitation wavelength of 360 nm and an emission wavelength of 460 nm. The AGEs inhibition percentage was calculated using the equation below:

Inhibition rate (%) = (1 − (intensity of test sample/intensity of control)) × 100%

Onemillilitreof MGO (60 mmol/L in 0.01 mol/L PBS, pH 7.4) solution was incubated with 100 μL of MK_8_(H_2_), MK4, or Q10 solution (2.5, 5, 10, 15, or 20 mg/mL) at 37 °C for 2 h, respectively. Acetone (100 μL) was used as a blank control. After 2 h incubation, 1 mL of the BSA solution (60 mg/mL in 0.01 mol/L PBS, pH 7.4) was added and incubated at 37 °C for six days. The measurements and calculation of AGEs inhibition were the same with the BSA-fructose model.

The arginine-MGO model was also performed to study the mechanism of AGEs production. Onemillilitre of MGO (60 mmol/L in 0.01 mol/L PBS, pH 7.4) solution was incubated with 100 μL of MK_8_(H_2_), MK_4_, or Q10 solution (2.5, 5, 10, 15, or 20 mg/mL) at 37 °C for 2 h, respectively. Acetone (100 μL) was used as a blank control. After 2 h incubation, 1 mL of the arginine solution (60 mg/mL in 0.01 mol/L PBS, pH 7.4) was added and incubated at 37 °C for six days. The measurements and calculation of AGEs inhibition were the same with the BSA-fructose model.

### 3.7. Determination of MK8(H2)-Carbonyl Adducts by LC-MS/MS

MK_8_(H_2_) (10 mg/L, 0.1 mL in acetone) was mixed with 0.5 mL of MGO (10 mmol/L, in 0.01 mol/L PBS, pH 7.4) and then incubated at 37 °C for 6 h. MK_8_(H_2_) with the PBS buffer solution at the same volume was served as a control. After incubation, 0.5 mL of o-phenylenediamine (50 mmol/L in acetone) was added to terminate the reaction. The HPLC analysis was performed to evaluate the reaction process of MK_8_(H_2_) and MGO. The HPLC analysis of MGO before and after reaction was performed according to the published article using a C18 column [33]. The HPLC analysis of MK_8_(H_2_) before and after reaction was performed according to the published article using a C30 column [20].

To analyze the reaction product of MK_8_(H_2_) and MGO, Agilent 1100 Series LC-MSD-Trap-XCT and GS-120-5-C18-A column (250 × 4.6, 5 μm) were used here. The elution program was based on the published article [33]. Injection Volume was set at 40 μL. The column temperature was set at 25 °C and the samples were detected at 315 nm. MS conditions: APCI ion source, positive ion mode, the ion source temperature: 280 °C, capillary voltage: 1500 V, drying air-flow rate: 5 L/min, atomizing chamber temperature: 280 °C, atomizing chamber voltage: 60 psi, the scanning range of mass-to-charge ratio: 30–2000.

### 3.8. Molecular Docking

The docking simulations were performed using Molecular Operating Environment (MOE), version 2018 (Chemical Computing Group, Montreal, QC, Canada). In this paper, the crystal structure of BSA was obtained from the RCSB Protein Data Bank (PDB code: 4F5S). Before docking, both the receptor and the ligand were prepared. The receptor (BSA)was processed to delete the original ligand and do the LigX operation. According to the published paper, typical residues (Trp212 (trptophan), Ser453 (serine), Arg217 (arginine), Val34 (valine), Ala341 (alanine), Try340 (trytophan), Asp450 (aspartic acid)) with a 4.5 Å extend were chosen as site I for the docking simulation. Similarly, typical residues (Trp134, Glu17 (glutamic acid), Gly21 (glycine), Gly135, Leu24 (leucine), Val40, Phe36 (phenylalanine), Asp129) with a 4.5 Å extend were chosen as site II for docking [34]. The structure of MK_8_(H_2_) as the ligand was built and energy-minimized by MOE. During docking, the following options should be set: for the first scoring function, choose “London dG” for Rescoring 1 and drop down its Retain option to 10; second, choose “Forcefield” for refinement; third, make sure the Rescoring 2 of the second scoring function is set to GBVI/WSA dG and Retain is set to 10. After docking, the conformation with the low S-score was chosen as the potential binding conformation.

### 3.9. Data Analysis

The antioxidation and antiglycation experiments were run in triplicate. The mean and standard deviation results were calculated, and inhibition curves were carried out using the Origin 8.0 software (Origin Lab, Northampton, MA, USA).

## Figures and Tables

**Table 1:** HPLC-DAD-MS/MS (diode array detector-mass spectrum) analysis of Menaquinones (MKs) in *Rhodococcus* sp. B7740.

Peak ^a^	Compound ^b^	Formula	t_R_ (min)	λ_max_ (nm) ^c^	[M + H]^+^ (*m*/*z*)	MS/MS (*m*/*z*)
1	MK_8_(H_2_)	C_51_H_74_O_2_	10.2	250,264	719	701,675,227,
2	MK_8_	C_51_H_72_O_2_	10.4	248,262	717	699,567,227
3	MK_8_(H_4_)	C_51_H_76_O_2_	13.0	252	721	703,571,227
4	MK_8_(H_6_)	C_51_H_78_O_2_	14.4	252	723	705,571,227
5	MK_7_(H_2_)	C_46_H_66_O_2_	15.4	243,268	651	633,567,187
6	MK_8_(H_2_)	C_51_H_74_O_2_	19.7	247,269	719	701,635,227
7	MK_9_(H_4_)	C_56_H_84_O_2_	24.8	223,269	789	770,637,501

^a^ The peak number is based on the chromatogram in Figure 1B; ^b^ Tentative identification with the combined information; ^c^ Linear gradient of elution solvents.

**Table 2:** ^1^Hnuclear magnetic resonance (NMR) and ^13^C NMR information of MK_8_(H_2_).

C/H Position	^1^H	^13^C	C/H Position	^1^H	^13^C
1		185.40	11′		124.28
2		143.43	12′	3.60–3.75 (m)	35.63
3		146.05	13′	3.60–3.75 (m)	32.31
4		184.50	14′	5.00 (t)	124.27
5	8.08 (q)	126.20	15′		118.84
6	7.68 (q)	134.87	16′	3.60–3.75 (m)	31.92
7	7.67 (q)	133.34	17′	0.91–1.20(m)	29.60
8	8.07 (q)	126.12	18′	0.91–1.20(m)	29.43
9		131.25	19′	1.50–2.09 (m)	29.38
10		132.17	20′	0.82–0.89 (m)	19.60
1′	3.38 (d)	70.7	21′	0.82–0.89 (m)	14.20
2′	5.34 (t)	129.35	2-CH_3_	2.19 (s)	29.72
3′		129.85	3′-CH_3_	0.90–1.48 (m)	29.34
4′	3.60–3.75 (m)	40.04	7′-CH_3_		27.25
5′	3.60–3.75 (m)	39.77	12′-CH_3_		26.71
6′	5.08–5.12 (m)	126.20	12′-CH_3_		26.02
7′		124.85	12′-CH_3_		25.71
8′	3.60–3.75 (m)	37.09	12′-CH_3_		25.47
9′	3.60–3.75 (m)	36.56	16′-CH_3_		25.26
10′	5.08–5.12 (m)	124.42			

## Supplementary Materials

The following are available online at http://www.mdpi.com/1660-3397/16/10/391/s1, Figure S1: HSCCC spectrum of Menaquinone from *Rhodococcus* sp. B7740 (A); ^1^H NMR (B), ^13^C NMR (C) and HSQC (D) spectra of MK_8_(H_2_), Figure S2: Antioxidant ability of MK_8_(H_2_) compared with Q10 and MK_4_ (A); Antiglycation effects of MK_8_(H_2_) compared with Q10 and MK_4_ in BSA-fructose model (B), BSA-MGO model (C) and arginine-MGO model (D).
