# Supplementary material for: Characterization of MK8(H2) from Rhodococcus sp. B7740 and Its Potential Antiglycation Capacity Measurements
Source: Mar Drugs. 2018 Oct 18;16(10):391. doi: 10.3390/md16100391 (PMC6213960; doi:10.3390/md16100391)
Supplement: Supplementary file 1 [file marinedrugs-16-00391-s001.pdf]

Figure S1

A

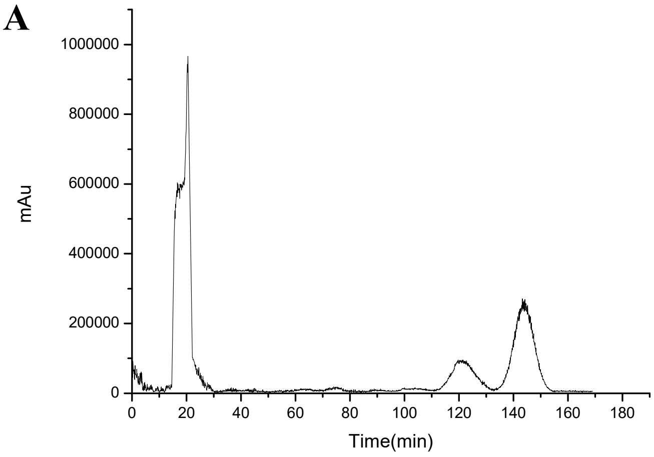
1000000
800000
600000
400000
200000
0
mAu
0
20
40
60
80
100
120
140
160
180
Time(min)

B

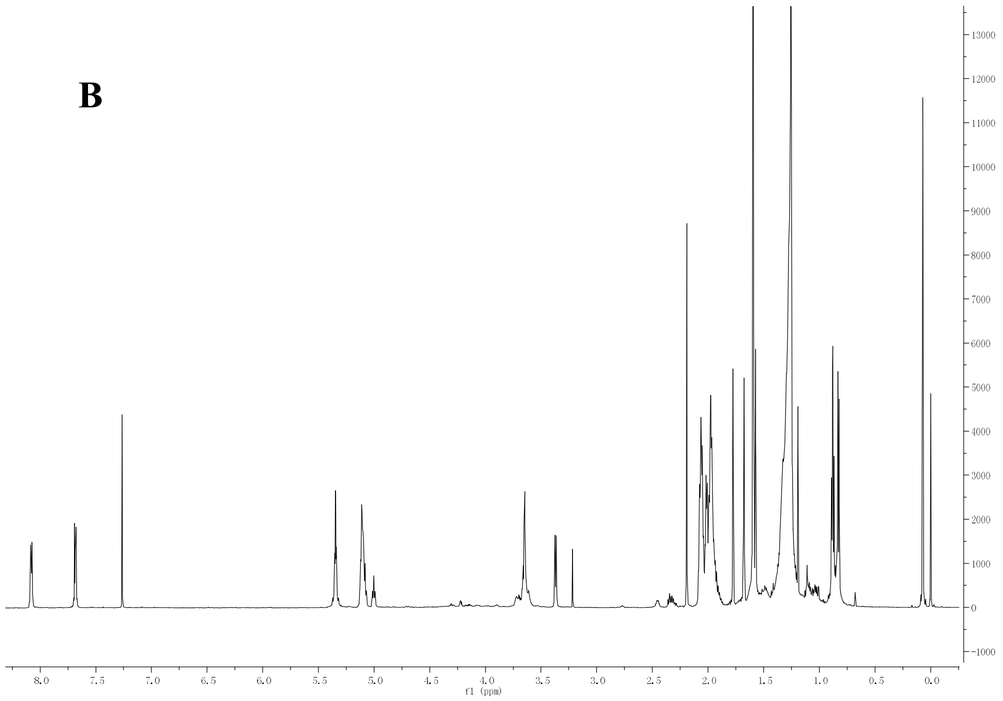
13000
12000
11000
10000
9000
8000
7000
6000
5000
4000
3000
2000
1000
0
−1000
8.0
7.5
7.0
6.5
6.0
5.5
5.0
4.5
4.0
3.5
3.0
2.5
2.0
1.5
1.0
0.5
0.0
f1 (ppm)

C

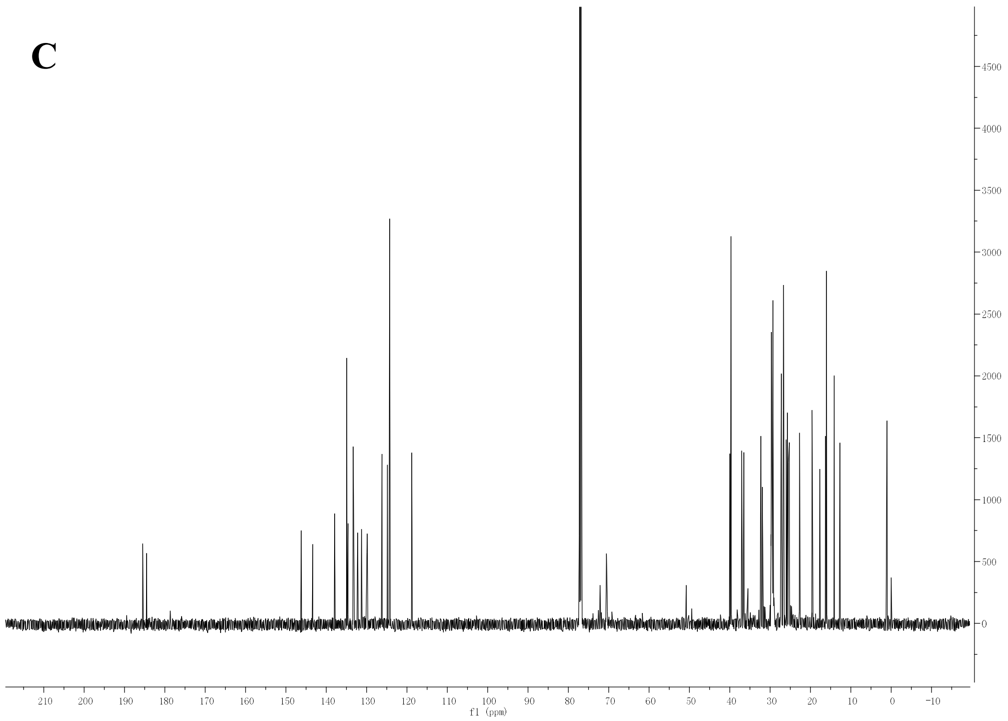

D

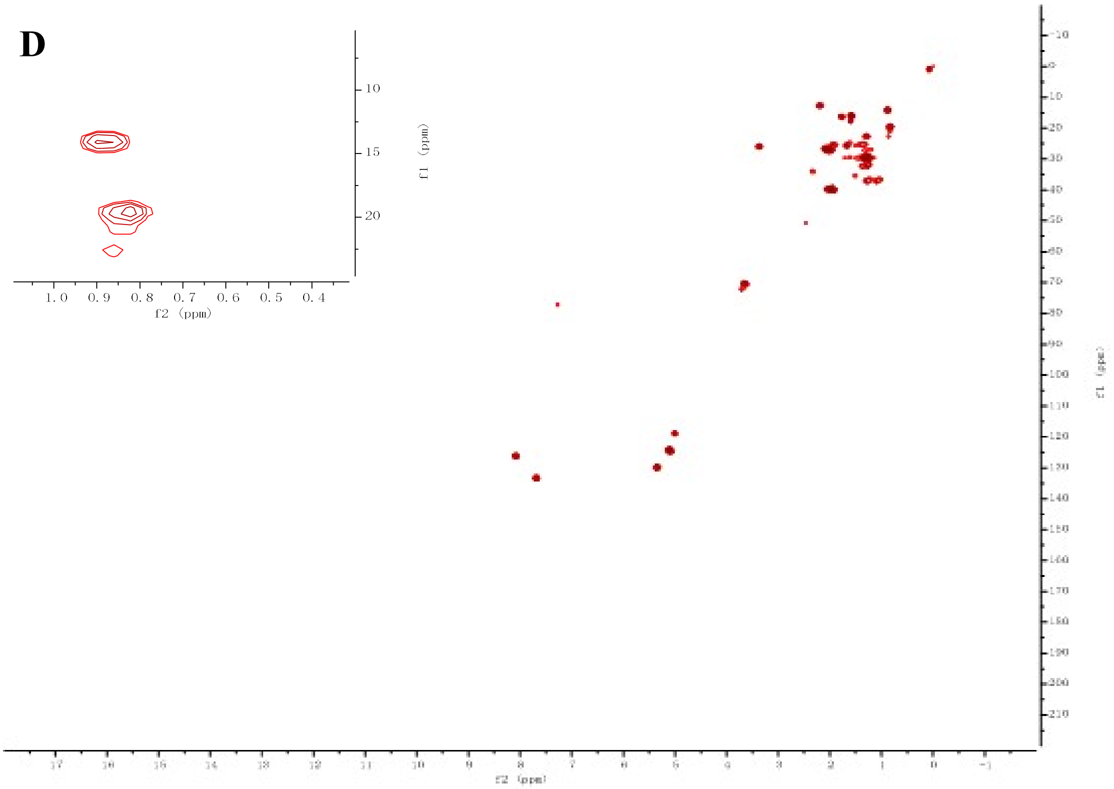

Figure S2

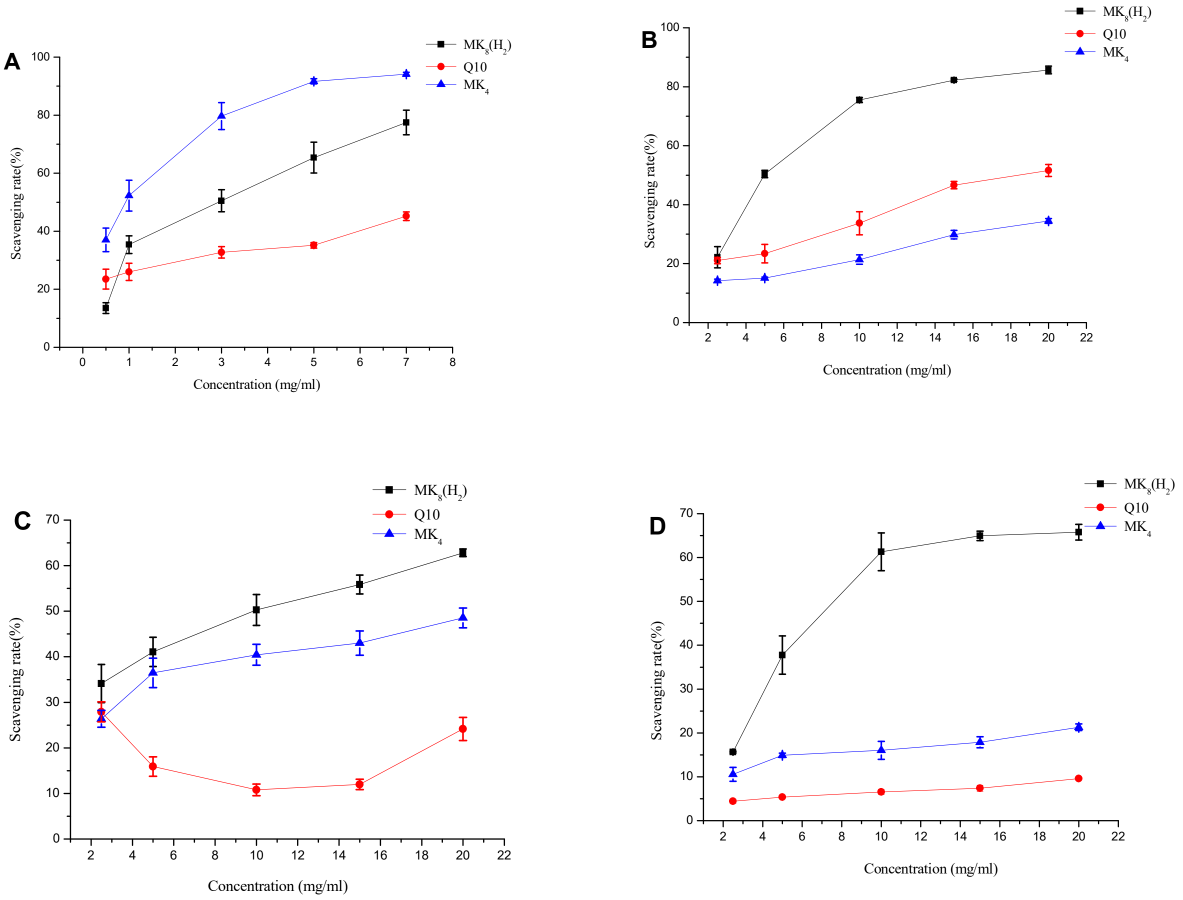

Figure S1. HSCCC spectrum of Menaquinone from Rhodococcus. sp B7740 (A); ^{1}HNMR (B), ^{13}CNMR (C) and HSQC (D) spectra of $MK_8(H_2)$.

Figure S2. Antioxidant ability of $MK_8(H_2)$ compared with Q10 and MK_4 (A); Antiglycation effects of $MK_8(H_2)$ compared with Q10 and MK_4 in BSA-fructose model (B), BSA-MGO model (C) and arginine-MGO model (D).
